# Nuclear Morphological Characteristics in Breast Cancer: Correlation with Hormone Receptor and Human Epidermal Growth Factor Receptor 2

**DOI:** 10.1155/2021/3037993

**Published:** 2021-11-12

**Authors:** Jiayu Li, Yehan Zhou, Yunzhu Li, Yang Liu

**Affiliations:** Department of Pathology, Sichuan Cancer Hospital & Institute, Sichuan Cancer Center, School of Medicine, University of Electronic Science and Technology of China, Chengdu, China

## Abstract

**Background:**

Hormone receptor (HR) and human epidermal growth factor receptor 2 (HER2) are the common diagnostic/prognostic markers in breast cancer. Few articles have recently reported the correlation between cytology and molecular subtypes. We combined nuclear morphological characteristics with HR and HER2 status to observe the relationship and provide ideas for machine learning.

**Methods:**

We reanalyzed fine-needle aspiration cytology samples and core-needle puncture histological specimens from 142 patients with invasive breast cancer between March 2019 and December 2019, and the findings were compared with the two groups (HR+/HER2- and HR-/HER2+) following nuclear cytomorphological features: nuclear/cytoplasmic ratio, difference of nuclear size, nuclear pleomorphism, chromatin feature, nuclear membrane and nucleoli, and Nottingham grading.

**Results:**

Two groups were significantly associated with the difference of nuclear size, nuclear pleomorphism, and nucleoli (*P* < 0.001) and consistent with histological grading (*P* < 0.001). Moreover, nucleolar characteristics of size and number had obviously statistical significance (*P* < 0.001). Multiple micro-nucleoli were frequently seen in the HR+/HER2- group compared with the HR-/HER2+ group which mostly were observed centered medium-large nucleoli. We described four interesting nuclear morphologies in the experiment.

**Conclusions:**

There were significant differences in nuclear characteristics between two groups. HR and HER2 status not only might be predicted in cytological samples, but some specific nuclear morphological features might have potential value to help us understand molecular function and predict more information.

## 1. Introduction

Breast cancer is a common malignant tumor in women, which usually involves heterogeneous expression of molecular markers. According to the molecular analysis of these markers, such as estrogen receptor (ER), progesterone receptor (PR), human epidermal growth factor receptor 2 (HER2), and Ki-67, breast cancer can be divided into luminal A, luminal B, HER2 overexpression, and basal-like subtypes that are related to different biological characteristics and treatments [1]. Currently, the immunohistochemical method instead of gene expression profile analysis in clinical work to identify these indicators has been widely recognized and applied all over the world.

Hormone receptor (HR) is the general term for ER and/or PR. HR and HER2 are both vital molecular markers involved in invasive breast cancer. ER, a member of the steroid hormone receptor superfamily, is mainly located at chromosome 6q24 in breast tissue and consists of 8 exons and 7 introns [2]. PR located downstream of the ER pathway shows the integrity of ER function [3]. As a predictor of endocrine therapy, HR expression may suggest a better prognosis than HR negative. HER2 is a member of the human epidermal growth factor (HER/EGFR/ERBB) family and located at 17q12, which encodes transmembrane glycoprotein of tyrosine kinase activity and plays an important role in regulating cell proliferation, differentiation, and growth [4]. HER2 overexpression is strongly associated with worse prognosis and becomes standard practice in the treatment of breast cancer which predict effective therapeutic targets to monoclonal antibodies directed against HER2 [5]. Therefore, these molecular indicators which can provide prognosis and predictive information of clinical management in breast pathology are of great importance.

Core needle biopsy (CNB) is a common diagnostic method for breast cancer, while fine-needle aspiration cytology (FNAC) is a convenient and fast method for pathological work which is commonly used in the differential diagnosis of benign and malignant breast diseases. Compared with histology, it can provide the complete structure of cells and morphological characteristics. Many scholars have done a lot of researches on the morphological features of breast tumor cells and found that the analysis of nuclear parameters is more meaningful [6]. Moreover, Cui et al. [7] have believed that the tumor nucleus is the main part that reflects the degree of differentiation and biological behavior and plays an increasingly important role in the study of tissue structure, cell morphology, and cytochemical quantification, as well as in assisting clinicopathological diagnosis and prognosis evaluation. The nuclear characteristics of FNAC and CNB were mainly described in our observation.

To the best of our knowledge, most studies have described the relationship between cytology and histological types of breast cancer, but few articles have recently reported the correlation between morphological characteristics and molecular subtypes. Here, combined morphological features with two representative groups of breast cancer, we analyzed the possible intrinsic correlation between cytology and molecular makers so that cytological characteristics may be explored to predict specific molecular changes and provide ideas for machine learning.

## 2. Materials and Methods

### 2.1. Patients and Samples

Cytological and histological specimens were, respectively, acquired by 22-G fine needles and 16-G core needles from 142 patients with immunohistochemically confirmed invasive breast cancer at Sichuan Cancer Hospital of Chengdu in China between March 2019 and December 2019. This study was approved by the hospital ethics committee. The criteria for our inclusion of cases are as followings: (1) sufficient tumor cells (more than the presence of 6 clusters of tumor cells with at least 10 cells in each cluster), (2) clear immunohisochemistry (IHC) and molecular analysis of invasive breast cancer with CNB, (3) two kinds of punctures at almost the same time, and (4) comprehensive and detailed clinical information, such as age, clinical stages, and histological types.

### 2.2. Molecular Analysis

Histological specimens by core needle biopsy were embedded in paraffin and stained with IHC. Although cytological specimens were not used for staining IHC directly, previous studies have shown that the expression of ER and HER2 performed on FNAC blocks was equivalent to that on histological specimens [8]. Immunohistochemical staining was performed by the EnVision method (Dako, Glostrup, Denmark) including ER (SP1, Maxin, Fuzhou, China), PR (SP2, Maxin), HER2 (EP3, ZSGB, Beijing, China), and Ki-67 (MIB-1, ZSGB).

We used American Society of Clinical Oncology/College of American Pathologists (ASCO/CAP) guidelines to define ER and PR with nuclear staining > 1% as expression [9]. ER or PR expression was defined as HR-positive (HR+). HER2 status was evaluated by ASCO/CAP guidelines that 3+ on immunohistochemical staining or HER2 amplification by fluorescence in situ hybridization (FISH) was considered HER2-positive (HER2+) [10]. In our study, the HR+/HER2- group is defined as ER and/or PR expression without HER2 expression, which belongs to the luminal subtype. In sharp contrast with the HR+/HER2- group, the HR-/HER2+ group is referred to HER2 overexpression subtype of only HER2 positive or gene amplification.

### 2.3. FNA and CNB Sample Evaluation

Tumor cells obtained from breast cancer were fixed in 95% ethanol and stained with Hematoxylin and Eosin staining (HE). Two independent observers who had been blinded to the subsequent clinical data and results of molecular markers reviewed the slices on a double-headed microscope, evaluating the smears with the nuclear morphological parameters (all data were found by visual differences and were consistent by simple quantitative determination) and the histological specimen with Nottingham grading (all data were found by visual differences) [11].

### 2.4. Statistical Analysis

The correlation between morphological features and two groups of invasive breast cancer was analyzed by Chi-square or Fisher exact test. Statistical analysis was performed using the Stata 13.0 software. A two-sided *P* value < 0.05 was considered statistically significant.

## 3. Results

### 3.1. Clinicopathological Characteristics

The baseline clinicopathologic features of 142 cases are presented in Table 1. All the patients were women aged 26-83 years (mean: 50.9 years) with unilateral lesions. According to the TNM pathological classification developed by the American Joint Committee on Cancer in 2010, there were 63 patients in stages I-II (44.4%) and 79 patients in stages III-IV (55.6%). As for the tumor size, we had the highest number of T2 patients (64.8%). Less than five lymph node metastases were most common seen in 111 of 142 cases (78.2%). Histologically, all the cases were invasive carcinoma of no specific type. There were no significant correlations between the two groups in clinicopathological characteristics.

### 3.2. Nuclear Morphological Features

As shown in Table 2, we observed the HR-/HER2- group in our experiment, but the results could be limited by the number of samples.

The HR+/HER2- and HR-/HER2+ groups were significantly associated with the difference of nuclear size and nuclear pleomorphism (*P* < 0.001). Besides, the greater difference of nuclear size (21/56, 37.5% vs. 2/86, 2.3%) and nuclear pleomorphism (9/56, 16.1% vs. 0/86, 0%) was more likely to the HR-/HER2+ group, while there was no statistical difference in nuclear/cytoplasmic ratio (*P* = 0.317) and chromatin feature (*P* = 0.202) between the HR+/HER2- group and the HR-/HER2+ group.

Moreover, the appearance of nucleoli was notably related to the two groups (*P* < 0.001) rather than the appearance of nuclear membrane (*P* = 0.314). We focused on describing the characteristics of the nucleoli including size and number. These nucleolar characteristics between the two groups also had obviously statistical significance (*P* < 0.001). Micro-nucleoli (average diameter ≤ 1 *μ*m) were frequently seen in the HR+/HER2- group (44/86, 51.2%) in contrast with the HR-/HER2+ group (9/56, 16.1%). On the contrary, medium-large nucleoli (average diameter > 1 *μ*m) of the HR-/HER2+ group (44/56, 78.6%) were more easily observed than that of the HR+/HER2- group (9/86, 10.5%). We also observed that most of the cases in the HR-/HER2+ group had one or two nucleoli (53/56, 94.6%) and were located in the relative middle position, while 37 cases (37/53, 70.0%) had commonly scattered multiple nucleoli except for 33 cases (33/86, 38.4%) without nucleoli in the HR+/HER2- group.

Just like the nuclear characteristics, there were correlations between histological grades in these two groups, especially about the pleomorphism and mitosis in Nottingham index (*P* < 0.001).

### 3.3. Internal Analysis of Each Group

According to the threshold of 20% for Ki-67 by St. Gallen Consensus, the HR+/HER2- group was divided into luminal A (Ki − 67 ≤ 20%) and luminal B (Ki − 67 > 20%) breast cancer [12]. The distribution of Ki-67 is shown in Table 3. The rate of Ki-67 high expression (Ki − 67 > 20%) was much higher in the HR-/HER2+ group (51/56, 91.1%) than that in the HR+/HER2- group (52/86, 60.5%). We found that the Ki-67 expression was related to nucleolar size (*P* < 0.001), nucleolar number (*P* = 0.003), histological grade (*P* = 0.022), and pleomorphism (*P* = 0.003). However, there were not correlated with any parameters in, respectively, the HR+/HER2- and HR-/HER2+ group. Then, we, respectively, studied the intensity (light-moderate, strength) and percentage (<50%, ≥50%) expression of ER in the HR+/HER2- group and the HER2 staining (2+, 3+) in the HR-/HER2+ group, and there were no significant differences in any parameters.

### 3.4. Special Images of Nuclear Morphology

**Figure 1:**
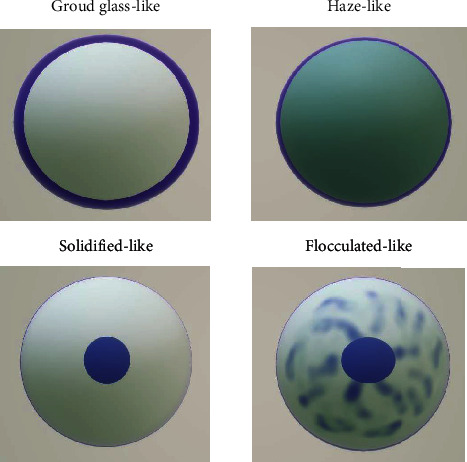
Patterns of four special nuclear morphologies.

**Figure 2:**
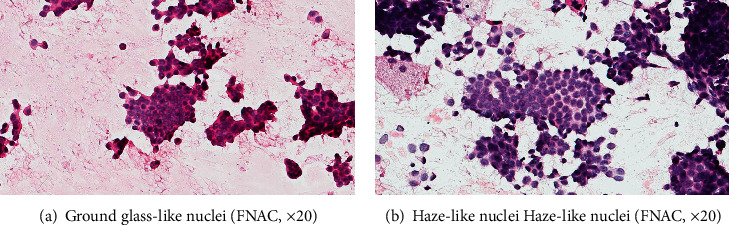
(a) Ground glass-like nuclei and (b) haze-like nuclei: nuclear membrane, light and homogeneous chromatin, micronucleoli (FNAC, ×20).

**Figure 3:**
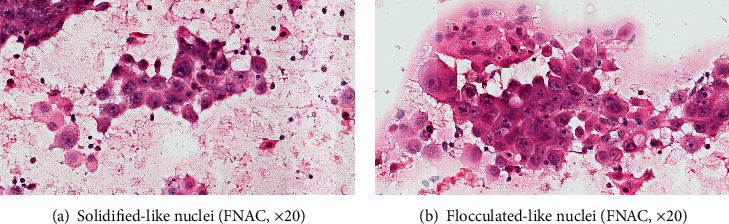
(a) Solidified-like nuclei and (b) flocculated-like nuclei: prominent nucleoli and halos around the nucleoli or heterogeneous chromatin (FNAC, ×20).

**Figure 4:**
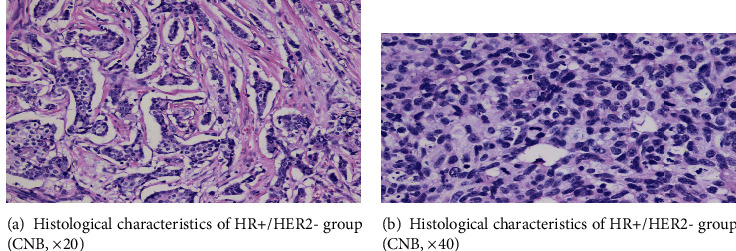
(a, b) Histological characteristics of HR+/HER2- group (CNB, ×20 and ×40).

**Figure 5:**
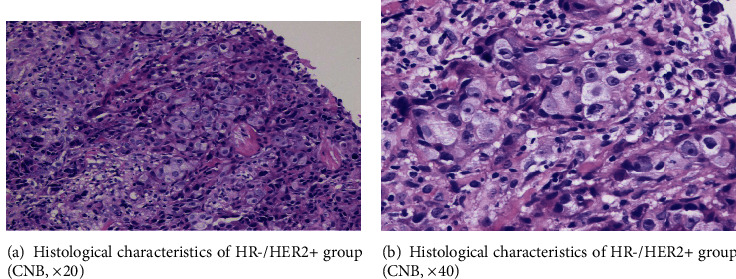
(a, b) Histological characteristics of HR-/HER2+ group (CNB, ×20 and ×40).

We observed four interesting nuclear morphological features in the experiment, and the pattern diagrams are shown in Figure 1. Ground glass-like nuclei similar to “Annie's eyes” often appeared in papillary thyroid carcinoma which have obviously nuclear membrane, light and homogeneous chromatin, inconspicuous, or micro-nucleoli (Figure 2(a)). Haze-like nuclei have darker chromatin and less clear nuclear membrane compared with ground glass-like nuclei (Figure 2(b)). Solidified-like nuclei are characterized by prominent nucleoli (Figure 3(a)). Flocculated-like nuclei have not only distinct nucleoli but heterogeneous or agglomerated chromatin (Figure 3(b)). The histological images (Figures 4(a) and 4(b)) corresponding to ground glass-like nuclei and haze-like nuclei had mild-moderate pleomorphism, while the histological images (Figures 5(a) and 5(b)) corresponding to solidified-like nuclei and flocculated-like nuclei had greater cellular atypia.

These four patterns were mainly described in the two groups (Table 4). Ground glass-like and haze-like nuclei were easier to find in the HR+/HER2- group (49/86, 57.0%) than in the HR-/HER2+ group (1/56, 1.8%), while solidified-like and flocculated-like nuclei accounted for a high proportion of the HR-/HER2+ group (24/56, 42.9%) in comparison with the HR+/HER2- group (6/86, 7.0%). Moreover, compared with ground glass-like nuclei, haze-like nuclei were more common in simultaneously strong expression of ER and PR.

## 4. Discussion

The correlation between tumor cytology, especially the cytomorphology of thyroid cancer and lung cancer, and its genes has already been described in previous literature, as well as these morphological features might be a valid tool for molecular analysis [13–17]. Nonetheless, there have been few detailed studies on the relationship between molecular markers and cytology in breast cancer. The aims of this study were to evaluate the specific morphological characteristics related to HR or HER2 status and to comprehend possible connections between them.

In our study, it was found that some nuclear morphological characteristics between the HR+/HER2- group and the HR-/HER2+ group were significantly different. Among nuclear appearances, HR or HER2 status was related to difference of nuclear size and nuclear pleomorphism (*P* < 0.001), although nuclear/cytoplasmic ratio and chromatin feature did not reach statistical significance in the two groups. It is well known that the nuclei are control towers of all the cell behaviors that turn out to be critical in cell migration, particularly in cancer invasion and metastasis [18]. The nuclear changes implicated in HR or HER2 that affect the occurrence and development of breast carcinoma may provide internal information. Our results were consistent with most studies that the size and pleomorphism of nuclei have played more important roles in breast cancer [6–7]. More specifically, the HER2 overexpression subtype had greater nuclear differences (37.5% vs. 2.3%) and larger nuclear pleomorphism (19.1% vs. 0%) than the HR+/HER2- group. Nuclear morphometry on cytological aspirates of breast cancer and its correlation with cytomorphological and histological grading have already been reported [19–20]. Consistent with other studies, HER2-positive breast cancer has some clinicopathological characteristics including high histological grade, large tumor size, and lymph node metastasis [21–22]. And Tamaki et al. [23] reported in more detail that the histological grade of the HR+/HER2- group was lower than that of the HR-/HER2+ group, which is compatible with our nuclear morphology. In general, HER2-positive breast cancer is more aggressive and has a higher risk of recurrence and metastasis than HER2-negative breast cancer [24–25]. The larger and more irregular nuclei tended to higher histological grade, and worse prognosis in breast cancer has been directly shown [26]. Therefore, it may explain the higher ratio of nuclear atypia in the HR-/HER2+ group than in the HR+/HER2- group.

In addition, the presence of nucleoli (*P* < 0.001) between the two groups was more statistically significant than the presence of nuclear membrane (*P* = 0.314). Nucleoli were observed more easily in the HR-/HER2+ group (53/56, 94.6%) than in the HR+/HER2- group (53/86, 61.6%). The nucleoli are highly dynamic structures that exhibit periodic disappearance and reconstruction during mitosis and carry out ribosomal RNA synthesis and ribosome formation. Hence, gen-related transcription and protein may be closely associated with nucleoli changes. Our study has been involved in two divergent molecular pathways of progression at the molecular level, mainly related to ER expression and tumor grade and proliferation [27]. ER exerts its biological effects mainly through the classic nuclear estrogen receptor pathway included ER*α* and ER*β* in spite of some reports on membrane estrogen receptor expression pathways in recent years. Estrogen-binding ER in the nucleus plays a slow “genotype” regulatory effect by regulating the transcription of specific target genes to activate intracellular signaling pathways, make cells respond, and promote cell proliferation and malignant transformation [2], while HER2 is the only receptor in ERBB family members without direct ligands. HER2 overexpression or the formation of heterodimers between HER2 and other family members may lead to the activation of HER2 pathway and affect various signaling pathway initiation, such as the RAS pathway and the phosphoinositide 3-kinase- (PI3K-) protein kinase B- (AKT-) mitogen-activated protein kinase (MAPK) pathway [28–29]. The differences of gene mechanisms between two groups may contribute to the differences of cell cycle and affect the appearance of nucleoli.

More nucleolar characteristics were also focused on describing statistically significant differences between the two groups. As for nucleolar size, the ratios of medium-large nucleoli in the HR-/HER2+ group and the HR+/HER2- group were 78.6% and 10.5%, respectively. Studies have shown that nucleolar size and cancer cell rRNA transcriptional activity were inversely related to cell doubling time which affected cell proliferation rate of cancer tissue [30–31]. That is, the medium-large nucleolar cells observed in the HR-/HER2+ group may proliferate faster than the small nucleolar cells in the HR+/HER2- group, and the related biological behavior and prognosis of HER2 overexpression subtype may be worse than only HR-positive group. There was a significant difference in nucleolar size between two groups, which corresponded with the clinicopathological features of the molecular changes of HR and HER2. Besides, we found that the HR+/HER2- group usually had inconspicuous nucleoli (33/86, 38.4%) or more than one micro-medium nucleoli (37/86, 43.0%), while the HR-/HER2+ group mostly had one nucleolus (49/53, 92.5%). Where protein synthesis is exuberant, there are large and many nucleoli. Ki-67, a significant indicator of cell proliferation, was also introduced and evaluated. Coinciding with our results, the Ki-67 expression was related to the number and size of nucleoli and histological grade. On the one hand, the Ki-67 high expression rate in the HR-/HER2+ group (51/56, 91.1%) was higher than in the HR+/HER2- group (52/86, 60.4%). It also indicated that the cell proliferation of HER2 overexpression subtype is more than that of only HR-positive group. In the internal study of each group, the nucleolar size of luminal B group had a larger trend than that of luminal A subtype, although there was no obviously statistical significance (*P* = 0.064). However, the Ki-67 expression was not related to any nuclear parameters in the HR-/HER2+ group. Based on our research, we believe that different genes lead to differences in cell proliferation, and the nucleolar size may better reflect cell proliferation than the number of nucleoli under the same molecular mechanism, like the HR+/HER2- group. Consequently, ER and HER2 are associated with different molecular pathways and the nuclear characteristics, especially the nucleolar features, more carefully showed the morphological similarities and differences between the two groups.

We mainly observed the typical images of four kinds of nuclear morphology on this basis. Ground glass-like or haze-like patterns were more common in luminal breast cancer (49/86, 57.0% vs. 1/56, 1.8%), and solidified-like or flocculated-like morphology of HER2 overexpression subtype had a higher ratio (24/56, 42.9% vs. 6/86, 7.0%), although these images can exist simultaneously in some cases. To put it simply, the HR+/HER2- group mostly had inconspicuous nucleoli or multiple scattered micro-nucleoli, while the HR-/HER2+ group usually had obvious one or two nucleoli that located in the relatively centered position. These were also the comprehensive embodiment of the morphological characteristics described above.

The ground glass-like nucleus of thyroid carcinoma is very similar to the ground glass-like or haze-like morphology we observed in breast cancer. The formation mechanism of this change is unknown and might be considered to be an artifact of fixation or embedding in thyroid carcinoma. Nevertheless, it has already been reported that their presence was considered to be a crucial characteristic of papillary thyroid carcinoma, which has been used as one of the main points of diagnosis so far [32]. There is a possibility that some features are true, and their presentation depends on different methods of processing or observation. Moreover, thyroid gland and breast are closely related to hormones, and the relationship between the two cancers has also been described in literature. Whether similar morphological characteristics indicate changes of connected genes or pathways is worthy of our discussion.

And then, the chromatin of solidified or flocculated-like morphology was different from ground glass and haze-like patterns. We found that most cases of the HR-/HER2+ group had heterogeneous and block chromatin, like solidified or flocculated-like morphology, and some cases had atypical large cells contained strongly chromatin in the HR-/HER2+ group. While a large number of HR+/HER2- cases had relatively homogeneous and fine chromatin of ground glass-like and haze-like patterns, few cases had fine chunks of heterogeneous chromatin. The DNA-containing chromosome has a loose spiral-like structure during the interphase of the cell cycle, while chromatin filaments are highly spiralized into different shapes such as thicker columns and rods during cell division. The impact of chromatin dynamics on gene expression and the critical role of chromatin in regulating transcription have already been reported [33–34]. It may also be related to different action mechanisms of distinct genes as mentioned above. Therefore, the proportions of the four images were different between the two groups as we have observed, and these typical morphologies may be clues for identification of HR or HER2 expression.

However, only two classical types of the HR+/HER2- and HR-/HER2+ groups were studied in our study. We additionally observed twenty-four cases of triple negative breast cancer and found some differences in nuclear characteristics and histological grade between the two groups. The observation results might be limited by the number of samples. We considered that triple-negative breast cancer (TNBC) has been defined as tumor that lack expression of ER, PR, and HER2. Actually, TNBC accounted for a small proportion of breast cancer is a highly heterogeneous group of tumors by some gene expression analysis [35]. So it may be due to the complexity of the molecules of triple negative breast cancer resulting in a variety of cell morphology. Theoretically, genes may be associated with different morphological changes which can reveal possibly inherent genetic status to some extent, but molecular pathways are interconnected and complicated. HR and HER2 are the more common molecular changes in breast cancer. There are many other genetic changes in the same tumor, and the same genetic mechanism can also act on different tumors. For example, BRCA1, BRCA2, TP53, CDH1, and PTEN have been studies in breast cancer [36–37], as well as HER2 has been identified in lung, gastric, and gynecological cancers [38]. Morphological changes are not necessarily specific, but they are bound to reflect changes of some genes and may show the comprehensive effects. For example, whether the HR+/HER2+ group contains common morphological features or comprehensive superposition of effects is worthy of our consideration.

In conclusion, we studied the nuclear morphology of invasive breast cancer between the HR+/HER2- group and the HR-/HER2+ group, which has hardly been reported in the previous literature. Moreover, our results have shown significant differences in nuclear morphological characteristics between the HR+/HER2- group and the HR-/HER2+ group, and we found four special morphologies that may have predictive significance. These nuclear morphologies can not only show its correlation with HR or HER2 status but also may have potential value to help us understand molecular function and predict more clinical information. At the same time, it also provided information for machine learning.

## Figures and Tables

**Table 1:** Patient characteristics.

Characteristics	HR+/HER2-	HR-/HER2+	All (*n* = 142)	*P* value
Age, median (range), years			50.9 (26-83)	0.193^∗^
≤50	48	25	73	
>50	38	31	69	
Sex				
Female			142	
Clinical stage				0.130
I	5	0	5	
II	36	22	58	
III	31	28	59	
IV	14	6	20	
Histological type				
Invasive carcinoma of no specific type			142	
Tumor size, cm				0.222
≤2	27	16	43	
2 < T ≤ 5	57	35	92	
> 5	2	5	7	
Number of metastic lymph nodes				0.637
< 5	68	43	111	
5 ≤ N < 10	15	9	24	
≥ 10	3	4	7	
Distant metastases				
Yes	8	2	10	0.316
None	78	54	132	

HR+/HER2-: hormone receptors-positive and human epidermal growth factor receptor 2-negative; HR-/HER2+: hormone receptors-negative and human epidermal growth factor receptor 2-positive. ^∗^By Chi-square test, others by Fisher exact test.

**Table 2:** Nuclear morphological characteristics of the invasive breast cancer with three groups.

Characteristics	Cases			*P* value		
	HR+/HER2-	HR-/HER2+	HR-/HER2-	HR+/HER2- vs. HR-/HER2+	HR+/HER2- vs. HR-/HER2-	HR-/HER2+ vs. HR-/HER2-
Nuclear/cytoplasmic ratio				0.317	0.610	1.000
≤1	82	51	22			
> 1	4	5	2			
Difference of nuclear size				<0.001	0.068	0.033
MAX/MIN ≤ 2	84	35	21			
MAX/MIN > 2	2	21	3			
Nuclear pleomorphism				<0.001	0.218	0.267
Mild-moderate	86	47	23			
Severe	0	9	1			
Chromatin feature				0.202	0.558	0.781^∗^
Fine-powder	82	42	19			
Granular	4	14	5			
Nuclear membrane				0.314^∗^	1.000^∗^	0.523^∗^
Absent or few	21	18	6			
Conspicuous	65	38	18			
Nucleoli				<0.001	0.053	0.189
Absent or few	33	3	4			
Conspicuous	53	53	20			
Nucleolar size				<0.001	<0.001	0.373
Micro	44	9	2			
Medium	9	36	12			
Large	0	8	6			
Nucleolar number				<0.001	<0.001	1.000
1	16	49	19			
2	18	4	1			
≥ 3	19	0	0			
Histological grade				<0.001	0.019	<0.001
I	19	0	0			
II	59	30	22			
III	8	26	2			
Tubule formation				0.075	<0.001	0.090
1	5	0	0			
2	19	19	13			
3	62	37	11			
Pleomorphism				<0.001	<0.001	<0.001
1	28	0	0			
2	54	20	20			
3	4	36	4			
Mitotic counts				<0.001	0.004	0.668
1	23	0	0			
2	57	50	23			
3	6	6	1			

Nucleolar size: micro (average diameter ≤ 1 *μ*m), medium (1 *μ*m < average diameter ≤ 2 *μ*m), and large (average diameter > 2 *μ*m).

**Table 3:** Nuclear morphological characteristics of the invasive breast cancer with Ki-67 expression.

Characteristics	HR+/HER2-			HR-/HER2+			All cases		
	Ki − 67 ≤ 20%	Ki − 67 > 20%	*P* value	Ki − 67 ≤ 20%	Ki − 67 > 20%	*P* value	Ki67 ≤ 20%	Ki − 67 > 20%	*P* value
	*N* = 34 (39.5%)	*N* = 52 (60.5%)		*N* = 5 (8.9%)	*N* = 51 (91.1%)		*N* = 39 (27.5%)	*N* = 103 (72.5%)	
Nuclear/cytoplasmic ratio			1.000			1.000			0.444
≤1	33	49		5	46		38	95	
> 1	1	3		0	5		1	8	
Difference of nuclear size			1.000			1.000			0.126
MAX/MIN ≤ 2	33	51		3	32		36	83	
MAX/MIN > 2	1	1		2	19		3	20	
Nuclear pleomorphism			1.000			0.580			0.064
Mild-moderate	34	48		5	46		39	94	
Severe	0	4		0	5		0	9	
Chromatin feature			1.000^∗^			0.316			0.244^∗^
Fine-powder	29	43		5	37		34	80	
Granular	5	9		0	14		5	23	
Nuclear membrane			0.504^∗^			0.652			0.533^∗^
Absent or few	7	14		2	16		9	30	
Conspicuous	27	38		3	35		30	73	
Nucleoli			0.635^∗^			0.249			0.179^∗^
Absent or little	12	21		1	2		13	23	
Conspicuous	22	31		4	49		26	80	
Nucleolar size			0.064			1.000			<0.001
Micro	21	22		1	8		22	30	
Medium	2	8		3	33		5	41	
Large	0	0		0	8		0	8	
Nucleolar number			0.632			1.000			0.003^∗^
1	4	9		4	44		8	53	
2	6	9		0	4		6	13	
≥ 3	12	13		0	1		12	14	
Histological grade			0.365			0.172			0.022^∗^
I	10	9		0	0		10	9	
II	22	37		1	29		23	66	
III	2	6		4	22		6	28	
Tubule formation			0.590			0.155			0.174
1	3	2		0	0		3	2	
2	8	11		0	19		8	30	
3	23	39		5	32		28	71	
Pleomorphism			0.462			0.645			0.003^∗^
1	14	14		0	0		14	14	
2	19	35		1	19		20	54	
3	1	3		4	32		5	35	
Mitotic counts			0.449			1.000			0.255
1	8	15		0	0		8	15	
2	25	32		5	45		30	77	
3	1	5		0	6		1	11	

**Table 4:** Special images of nuclear morphology.

Image	HR+/HER2- (*n* = 86) %		HR-/HER2 + (*n* = 56) %	
Inconspicuous nucleoli group	49	57.0	1	1.8
Ground-glass	38	44.2	1	1.8
Haze	11	12.8	0	0
Distinct nucleoli group	6	7.0	24	42.9
Solidified	0	0	17	30.4
Flocculated	6	7.0	7	12.5

## Data Availability

Cytological and histological specimens were, respectively, acquired by 22-G fine needles and 16-G core needles from 142 patients with immunohistochemically confirmed invasive breast cancer at Sichuan Cancer Hospital of Chengdu in China between March 2019 and December 2019. This study was approved by the hospital ethics committee.

## Additional Points

*Home Messages*. We studied the nuclear morphology of invasive breast cancer between the HR+/HER2- group and the HR-/HER2+ group. There were significant differences in nuclear morphological characteristics with HR and HER2 status. And we found four special morphologies that may have potential value to help us understand molecular function and predict more clinical information. At the same time, it also provided information for machine learning.

## References

[1] Sørlie T., Tibshirani R., Parker J. (2003). Repeated observation of breast tumor subtypes in independent gene expression data sets. *Proceedings of the National Academy of Sciences of the United States of America*.

[2] Prossnitz E. R., Barton M. (2014). Estrogen biology: new insights into GPER function and clinical opportunities. *Molecular and Cellular Endocrinology*.

[3] Forster C., Makela S., Warri A. (2002). Involvement of estrogen receptor beta in terminal differentiation of mammary gland epithelium. *Proceedings of the National Academy of Sciences of the United States of America*.

[4] Klapper L. N., Glathe S., Vaisman N. (1999). The ErbB-2/HER2 oncoprotein of human carcinomas may function solely as a shared coreceptor for multiple stroma-derived growth factors. *Proceedings of the National Academy of Sciences of the United States of America*.

[5] Bauer K. R., Brown M., Cress R. D., Parise C. A., Caggiano V. (2007). Descriptive analysis of estrogen receptor (ER)-negative, progesterone receptor (PR)-negative, and HER2-negative invasive breast cancer, the so-called triple-negative phenotype. *Cancer-Am Cancer Soc.*.

[6] Howell L. P., Lin-Chang L. (2005). Cytomorphology of common malignant tumors of the breast. *Clinics in Laboratory Medicine*.

[7] Cui Y., Koop E. A., van Diest P. J., Kandel R. A., Rohan T. E. (2007). Nuclear morphometric features in benign breast tissue and risk of subsequent breast cancer. *Breast Cancer Research and Treatment*.

[8] Vohra P., Buelow B., Chen Y.-Y. (2016). Estrogen receptor, progesterone receptor, and human epidermal growth factor receptor 2 expression in breast cancer FNA cell blocks and paired histologic specimens: a large retrospective study. *Cancer Cytopathology*.

[9] Hammond M. E. H., Hayes D. F., Wolff A. C., Mangu P. B., Temin S. (2010). American society of clinical oncology/college of american pathologists guideline recommendations for immunohistochemical testing of estrogen and progesterone receptors in breast cancer. *Journal of Oncology Practice/ American Society of Clinical Oncology*.

[10] Press M. F., Sauter G., Buyse M. (2016). HER2 gene amplification testing by fluorescent in situ hybridization (FISH): comparison of the ASCO-College of American Pathologists Guidelines with FISH scores used for enrollment in Breast Cancer International Research Group clinical trials. *Journal of Clinical Oncology*.

[11] Pujani M., Sharma K. L., Srivastava A. N., Singh U. S., Bansal C. (2014). Grading systems in the cytological diagnosis of breast cancer: a review. *Journal of Cancer Research and Therapeutics*.

[12] Urruticoechea A., Smith I. E., Dowsett M. (2005). Proliferation marker Ki-67 in early breast cancer. *Journal of Clinical Oncology*.

[13] Klijanienko J., Pierron G., Sastre-Garau X., Theocharis S. (2015). Value of combined cytology and molecular information in the diagnosis of soft tissue tumors. *Cancer Cytopathology*.

[14] Rossi E. D., Bizzarro T., Martini M. (2014). Morphological parameters able to predictBRAFV600E-mutated malignancies on thyroid fine-needle aspiration cytology: our institutional experience. *Cancer Cytopathology*.

[15] Shahi M., Bloechl S. J., Vogel R. I. (2019). Semiquantitative assessment of cytomorphologic features can predict mutation status of thyroid nodules with indeterminate cytologic diagnosis. *Human Pathology*.

[16] Nishino M., Klepeis V. E., Yeap B. Y. (2012). Histologic and cytomorphologic features of _ALK-_ rearranged lung adenocarcinomas. *Modern pathology: an official journal of the United States and Canadian Academy of Pathology, Inc.*.

[17] Marotti J. D., Schwab M. C., McNulty N. J. (2013). Cytomorphologic features of advanced lung adenocarcinomas tested for EGFR and KRAS mutations: a retrospective review of 50 cases. *Diagnostic Cytopathology*.

[18] Kim D. H., Hah J., Wirtz D. (2018). Mechanics of the cell nucleus. *Advances in Experimental Medicine and Biology*.

[19] Moroz K., Lipscomb J., Vial L. J., Dhurandhar N. (1997). Cytologic nuclear grade of malignant breast aspirates as a predictor of histologic grade. Light microscopy and image analysis characteristics. *Acta Cytologica*.

[20] Kashyap A., Jain M., Shukla S., Andley M. (2018). Role of nuclear morphometry in breast cancer and its correlation with cytomorphological grading of breast cancer: a study of 64 cases. *Journal of Cytology*.

[21] Alnegheimish N. A., Alshatwi R. A., Alhefdhi R. M., Arafah M. M., AlRikabi A. C., Husain S. (2016). Molecular subtypes of breast carcinoma in Saudi Arabia. A retrospective study. *Saudi Medical Journal*.

[22] Zavyalova M., Vtorushin S., Telegina N. (2016). Clinicopathological features of nonspecific invasive breast cancer according to its molecular subtypes. *Experimental Oncology*.

[23] Tamaki M., Kamio T., Kameoka S., Kojimahara N., Nishikawa T. (2013). The relevance of the intrinsic subtype to the clinicopathological features and biomarkers in Japanese breast cancer patients. *World Journal of Surgical Oncology*.

[24] Loibl S., Gianni L. (2017). HER2-positive breast cancer. *The Lancet.*.

[25] Sotiriou C., Pusztai L. (2009). Gene-expression signatures in breast cancer. *The New England Journal of Medicine*.

[26] Abdalla F., Boder J., Markus R. (2009). Correlation of nuclear morphometry of breast cancer in histological sections with clinicopathological features and prognosis. *Anticancer Research*.

[27] Harbeck N., Penault-Llorca F., Cortes J. (2019). Breast cancer. *Nature Reviews. Disease Primers*.

[28] Cortés J., Saura C., Bellet M. (2011). HER2 and hormone receptor-positive breast cancer--blocking the right target. *Nature Reviews. Clinical Oncology*.

[29] Elster N., Collins D. M., Toomey S., Crown J., Eustace A. J., Hennessy B. T. (2015). HER2-family signalling mechanisms, clinical implications and targeting in breast cancer. *Breast Cancer Res Tr.*.

[30] Derenzini M., Trere D., Pession A. (1998). Nucleolar function and size in cancer cells. *The American Journal of Pathology*.

[31] Derenzini M., Trerè D., Pession A. (2000). Nucleolar size indicates the rapidity of cell proliferation in cancer tissues. *The Journal of Pathology.*.

[32] Kiyono T., Katagiri M., Harada T. (1994). The incidence of ground glass nuclei in thyroid diseases. *Thyroidology*.

[33] Hubner M. R., Spector D. L. (2010). Chromatin dynamics. *Annual Review of Biophysics*.

[34] Vaquerizas J. M., Akhtar A., Luscombe N. M. (2011). Large-scale nuclear architecture and transcriptional control. *Sub-Cellular Biochemistry*.

[35] Denkert C., Liedtke C., Tutt A., von Minckwitz G. (2017). Molecular alterations in triple-negative breast cancer--the road to new treatment strategies. *The Lancet.*.

[36] Banerji S., Cibulskis K., Rangel-Escareno C. (2012). Sequence analysis of mutations and translocations across breast cancer subtypes. *Nature*.

[37] Kothari C., Diorio C., Durocher F. (2020). Gene signatures of breast cancer development and the potential for novel targeted treatments. *Pharmacogenomics*.

[38] Ding Q., Chen H., Lim B. (2019). _HER2_ somatic mutation analysis in breast cancer: correlation with clinicopathological features. *Human Pathology*.

